# Nicotinamide, a vitamin B3 ameliorates depressive behaviors independent of SIRT1 activity in mice

**DOI:** 10.1186/s13041-020-00703-4

**Published:** 2020-11-23

**Authors:** Zhuxi Liu, Caiqin Li, Xuelian Fan, Yifang Kuang, Xu Zhang, Lei Chen, Jinjing Song, Ying Zhou, Eiki Takahashi, Guang He, Weidong Li

**Affiliations:** Bio-X Institutes, Key Laboratory for the Genetics of Development and Neuropsychiatric Disorders (Ministry of Education), Shanghai Key Laboratory of Psychotic Disorders, and Brain Science and Technology Research Center, Institute of Psychology and Behavioral Sciences, Shanghai Jiao Tong University, Shanghai, 200240 China

**Keywords:** SIRT1, Nicotinamide, ATP, Restraint animal model, Depression

## Abstract

Sirtuin 1 (SIRT1), is a nicotinamide adenine dinucleotide (NAD^+^)-dependent protein deacetylase and a candidate gene for depression. Nicotinamide (NAM), a form of vitamin B3, is reported as a potential inhibitor of SIRT1. Our previous study found that the 24-h-restraint stress could induce long-term depressive-like phenotypes in mice. These mice displayed increased SIRT1 activity. Here, we studied whether NAM was capable of attenuating depressive behaviors through inhibiting SIRT1 activity. Surprisingly, the application of NAM significantly reversed the depressive behaviors but increased SIRT1 activity further. In contrast, the level of adenosine triphosphate (ATP) was reduced in the restraint model for depression, and recovered by the administration of NAM. Furthermore, the *Sirt1*^*flox/flox*^*; Nestin-Cre* mice exhibited antidepressant behaviors and increased ATP levels. These data suggest that ATP plays an important role in depression pathogenesis, and NAM could be a potential treatment method for depression by regulating ATP independent of SIRT1 activity.

## Introduction

Depression is a common mental disorder accompanied by several psychological and emotional symptoms, and it affects approximately 4.4% of the world’s population [1]; however, the complex mechanisms underlying the pathogenesis of depression remain unclearly. Sirtuin 1 (SIRT1) is a protein deacetylase that contribute to cellular regulation in vivo. Studies have suggested it is associated with depression [2–4]. However, whether its increase or decrease contributing to depressive phenotypes is controversial in animal modeling studies. Abe-Higuchi et al. reported that SIRT1 activity in the dentate gyrus (DG) of the hippocampus was reduced by chronic stress in mice [5], where Ferland et al. demonstrated that chronic stress exposure enhances SIRT1 activity in the DG of rats [6]. Kim et al. demonstrated that stress induced SIRT1 expression in the nucleus accumbens (NAc) and altering SIRT1 activity could regulate anxiety- and depressive-like behaviors [7]. Besides, SIRT1 overexpress mice were more susceptible to depression compared to their wildtype littermates [8]. According to our previous study, we constructed the 24-h-restraint model with long lasting depressive-like phenotypes [9]. In this model of depression, we found that SIRT1 activity was increased.

Nicotinamide (NAM), a form of Vitamin B3, has been suggested to be therapeutically effective against many diseases and conditions, and it is mainly applied to pellagra in the clinics. Evidence has also suggested that NAM aids recovery from depression or bipolar disorders [10, 11]. Song et al. considered NAM to primarily work through increasing and decreasing monoamine-neurotransmitter synthesis and degradation respectively; they also considered NAM to potentially ameliorate depression through an antioxidative effect along with an increasing supply of nicotinamide adenine dinucleotide (NAD^+^) [12]. According to previous reports, NAM, which is produced by sirtuin enzymes, can inhibit the deacetylation of SIRT1 by binding to a conserved pocket adjacent to NAD^+^, thereby altering the NAD^+^ co-substrate specificity of a Sir2 enzyme [13, 14].

Then, we investigated whether NAM could attenuate depressive behaviors through inhibiting SIRT1 activity in 24-h-restraint mouse model. Strikingly, our result showed that SIRT1 activity was further increased after the administration of NAM, along with the fully rescue of depressive behaviors in mice.

## Methods

### Animals

Adult male C57BL/6 mice (age 12 weeks old) were obtained from Beijing Vital River Laboratory Animal Technology Co., Ltd, which were housed in groups per cage in a temperature-controlled room with a standard 12-h light/12-h dark cycle (light on from 7:00 a.m. to 7:00 p.m., 22 ℃ ± 2 ℃). We generated conditional *Sirt1*-deleted mice by crossing *Sirt1*^*flox/flox*^ mice (from Jackson Lab, 008041) with Nestin-Cre mice. The *Sirt1*^*flox/flox*^ mice were in a mixed 129SvJ/C57B6 background, while the *Nestin-Cre* mice were C57BL/6. The resulting *Sirt1*^*flox/*+^*; Nestin-Cre* mice were mated with *Sirt1*^*flox/flox*^ mice to obtain conditional *Sirt1-*knockout mice (*Sirt1*^*flox/flox*^*; Nestin-Cre*), while the corresponding *Sirt1*^*flox/flox*^ mice were used as the control. All animal experiments were performed according to the guidelines approved by the University Committee on Animal Care and Use of Shanghai Jiao Tong University, China.

### Animal model of the 24-h restraint

The experimental procedure was performed according to the protocol described previously in our laboratory with slightly modifications [9]. The mice were placed in a ventilated clear plastic tube (3 cm in diameter and 10 cm in length) and subjected to 24-h restraint from 14:00 p.m. to 14:00 p.m. of the next day. Once the restraint ended, the mice were returned to their home cages with access to food and water ad libitum. The control group remained in their home cages without the 24-h-restraint procedure.

### Drug administration

NAM reagent (Beyotime, Shanghai, China) was dissolved in drinking water at a 200 mg/kg/day dose [15, 16]. NAM treatment lasted for 33 days, beginning 2 days after the end of the 24-h restraint, whereas control mice were provided with sufficient water. The solutions were changed every 3 days until the mice were sacrificed.

### Behavioral procedures

After 5 weeks of 24-h restraint, behavioral tests were performed to verify the validity of the model to further study the mechanism of depression.

### Sucrose preference test (SPT)

During the adaptation period, mice were individually housed to habituation with two bottles containing either 2% sucrose (Sigma-Aldrich) diluted in drinking water or drinking water alone. The habituation was sustained for 3 days with the positions of the two bottles exchanged every 24 h. Water was removed at 4:00 p.m. for 17 h, and the test was started at 10:00 a.m. the next day. The test was performed for a total of 24 h, and then the positions of the two bottles were exchanged 12 h later.

### Forced swim test (FST)

The FST, which measures acute stress responses, was performed during the light phase (10:00–17:00). Mice were placed in a 2 L beaker containing water (24 °C ± 1 °C) for 5 min in a dim environment. The animals were analyzed for time spent on immobility.

### Western blot

Hippocampus tissues were isolated and lysed in RIPA buffer supplemented with protease inhibitor cocktail (Roche). After centrifugation (12,000*g*, for 20 min at 4 °C), the supernatants were retained and quantified using a BCA protein assay kit (Beyotime, Shanghai, China). Equivalent proteins were subjected to 10% SDS–PAGE and then transferred electrophoretically to polyvinylidene fluoride (PVDF) membranes at 300 mA for 60 min. After blocking with 5% bovine serum albumin (BSA), membranes were incubated with primary antibodies overnight at 4 °C, namely Sirt1 (1:1000, abcam) and β-actin (1:5000, CST). After incubation with appropriate horseradish peroxidase-conjugated secondary antibody (Millipore, USA), a high-sensitivity ECL reagent (Share-bio, China) was used. All the bands were analyzed with Image J.

### Quantitative real-time polymerase chain reaction

Total RNA was extracted from the hippocampus using triazole (Invitrogen) reagent, and then real-time polymerase chain reaction (RT-PCR) amplification and sequencing were performed. The cDNA was synthesized using reagents from a reverse transcription kit (Takara) per manufacturer's instructions. Quantitative PCR (qPCR) was performed using an SYBR Green 5 × PCR Master Mix (Takara) in an RT-PCR system performed on a Light Cycler 480 II (Roche). The primers are listed as follows: SIRT1-F: 5′-GCTGACGACTTCGACGACG-3′, SIRT1-R: 5′-TCGGTCAACAGGAGGTTGTCT-3′; GAPDH-F: 5′-TGACGTGCCGCCTGGAGAAAC-3′, GAPDH-R: 5′-CCGGCATCGAAGGTGGAAGAG-3’.

### SIRT1 activity assay

To measure SIRT1 activity, the protein was extracted from tissue using nondenaturing lysates, and protein concentrations were measured with a BCA protein assay kit (Beyotime, China). SIRT1 activity was quantified with a SIRT1 fluorometric assay kit (Sigma, CS1040) per manufacturer instructions. In brief, the reaction was conducted at 37 °C for 30 min, and deacetylase activity was detected and measured using a multimode reader (Tecan Infinite Pro, Switzerland; excitation wavelength = 360 nm, emission wavelength = 450 nm) [17].

### ATP assay

ATP levels of the hippocampus in mice were measured using a firefly luciferase-based ATP assay kit (Beyotime, Shanghai, China) per manufacturer instructions. Briefly, the tissue was lysed completely and centrifuged at 12,000*g* for 5 min at 4 ℃. After the background ATP had been consumed by 100 µL ATP detection working solution in a black 96-well plate, 20 µL of each supernatant were added to each well and assayed by a multimode reader (Tecan Infinite Pro, Switzerland).

### Statistical analysis

For all experiments, data were presented in terms of the mean ± standard error of the mean (SEM) and analyzed using GraphPad Prism software. An unpaired t test was used to determine the statistical differences between the two groups, and one-way analysis of variance (ANOVA) was used to analyze the variance for three groups. Given a significant effect of the one-way ANOVA, further multiple comparisons were conducted with Turkey-Kramer tests: *p* values < 0.05 were considered statistically significant.

## Results

### The SIRT1 activity is increased in the 24-h-restraint depressive mice

**Fig. 1:**
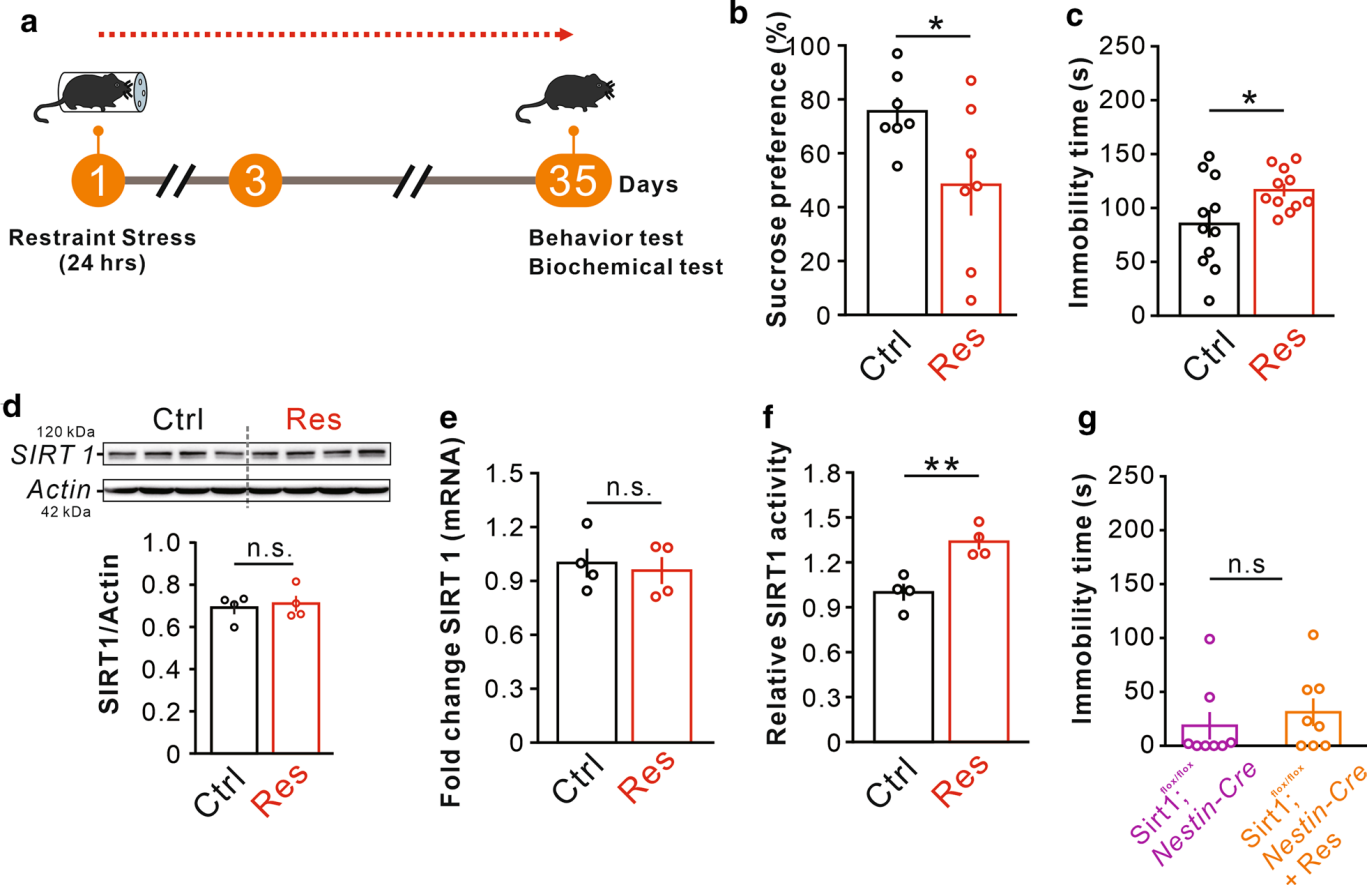
The 24-h-restraint stress increased SIRT1 activity in hippocampus. **a** 24-h-restraint mice were subjected to acute restraint for 24 h and used for behavioral or biochemical experiments 5 weeks later. **b** Decreased the long-term depressive-like behaviors of SPT (n = 7 per group), *p < 0.05. **c** Increased immobility time in restraint mice in the forced swimming test (n = 11 per group); *p < 0.05. **d** No difference in levels of SIRT1 (n = 4 per group). **e** Levels of SIRT1 mRNA in the hippocampus (n = 4 per group). **f** Increased SIRT1 activity in restraint group than that in control group (n = 4 per group); **p < 0.01. **g** The duration of immobility time in forced swim test was no significant change in the conditional *Sirt1* KO mice after 24-h-restraint stress (n = 8 per group). Data are presented as mean ± SEM. *Ctrl* control, *Res* restraint, *n.s.* no significance

Consistent with our previous report, the 24-h-restraint stress could produce long-term depressive-like phenotypes including deficits in sucrose preference test and forced swim test (Fig. 1a–c). To investigate the expression of SIRT1, we harvested hippocampal samples from 24-h-restraint (Res) and control (Con) mice 5 weeks after the modeling procedure. The qPCR and Western blot experiments were conducted. The protein and RNA levels of SIRT1 did not change in the hippocampus (Fig. 1d, e). However, SIRT1 activity was remarkably higher in the restraint mice than that in the control mice (Fig. 1f). As previous research reported that the brain-special conditional *Sirt1 *knockout mice displayed antidepressant behaviors [8], we subjected the mice to the 24-h-restraint stress and found that *Sirt1*^*flox/flox*^*; Nestin-Cre* mice were resistant to this stress in FST (Fig. 1g). These results suggested us that SIRT1 may play an important role in depression.

### Nicotinamide rescues depressive-like behaviors without inhibiting SIRT1 but increasing ATP

**Fig. 2:**
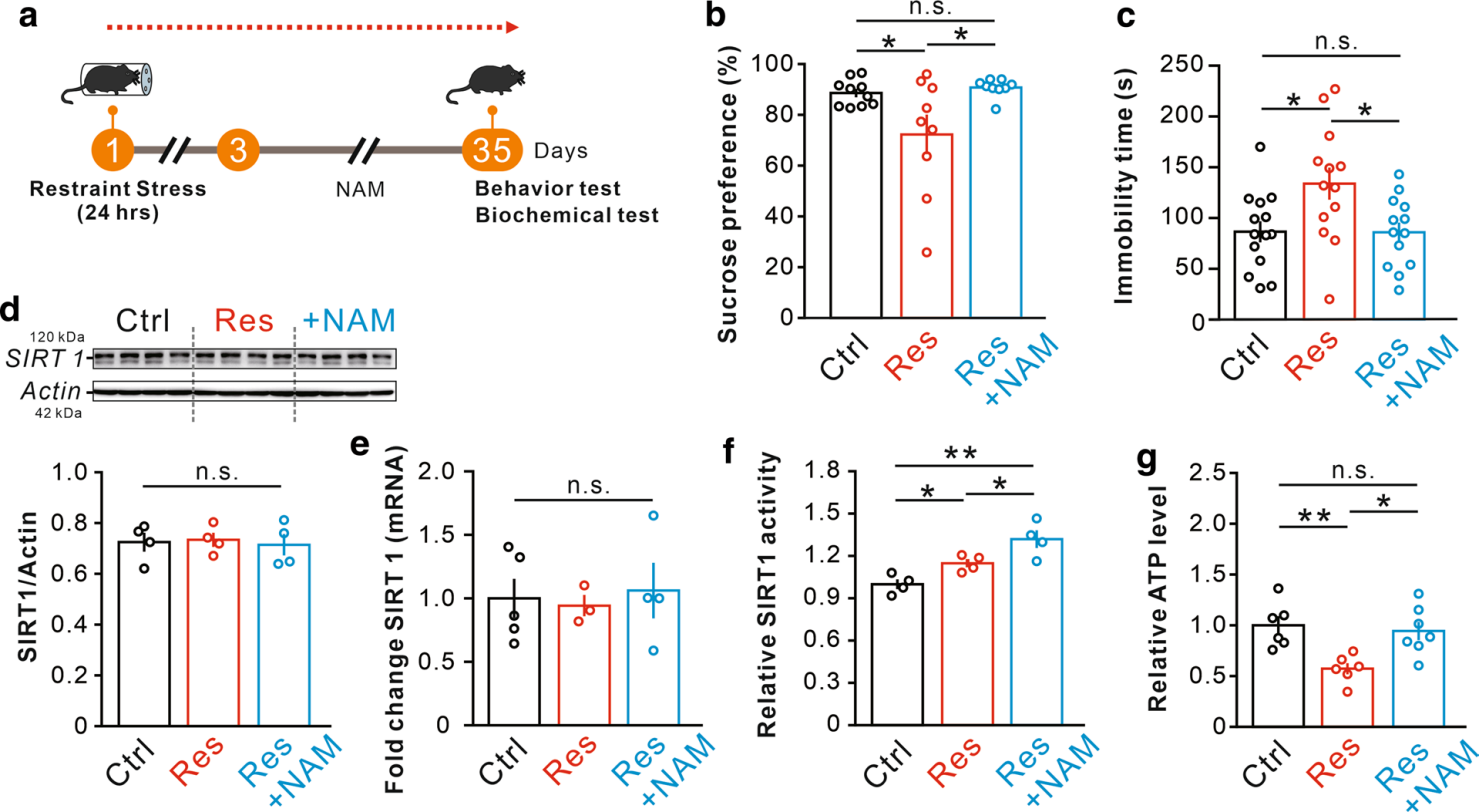
Nicotinamide could rescue the depressive-like behaviors induced by 24-h restraint. **a** NAM was administered 2 days after the restraint for the previous 5 weeks. **b** Sucrose consumption of three groups in the sucrose preference test (n = 10/9/9 per group); *p < 0.05. **c** Immobility time in the forced swim test (n = 14/13/13 per group); *p < 0.05. **d**, **e** Levels of SIRT1 protein and mRNA in the hippocampus (n = 4 per group). **f** The change of SIRT1 activity in the hippocampus of mice by NAM treatment (n = 4 per group); *p < 0.05, **p < 0.01. **g** The relative of ATP level in the hippocampus of mice (n = 6/6/7 per group); *p < 0.05, **p < 0.01

Based on this finding, we chose NAM, an inhibitor of SIRT1, to investigate whether inhibiting SIRT1 activity could alleviate depressive phenotypes. Mice were treated with NAM for 5 weeks since 2 days after the 24-h restraint and conducted similar behavioral tests mentioned above. The experimental process is illustrated in a schematic in Fig. 2a. The sucrose preference of the restraint mice was significant enhanced after NAM treatment (Fig. 2b). The duration of immobility time in forced swim test was significantly declined in the restraint mice with NAM (Fig. 2c). NAM could not change the expression of SIRT1 or RNA (Fig. 2d, e). However, SIRT1 activity was further increased by NAM (Fig. 2f). Therefore, we hypothesized that NAM might play a role in rescue depression through other ways. It was reported that NAM was the precursor of NAD^+^, an essential co-enzyme of redox reactions for ATP production [12]. There were also evidences showing that the depression was associated with a decrease of ATP [18, 19]. As our previous study found ATP was reduced in the 24-h-restraint depressive mice [9], we decided to detect the ATP level after NAM administration. Interestingly, we found that NAM could effectively reverse the ATP reduction caused by restraint stress in the hippocampus of the mice (Fig. 2g). These data suggested that ATP, instead of SIRT1 activity, plays a crucial role in regulating depression.

### The conditional *Sirt1* knockout mice exhibit antidepressant behaviors and increased ATP levels

**Fig. 3:**
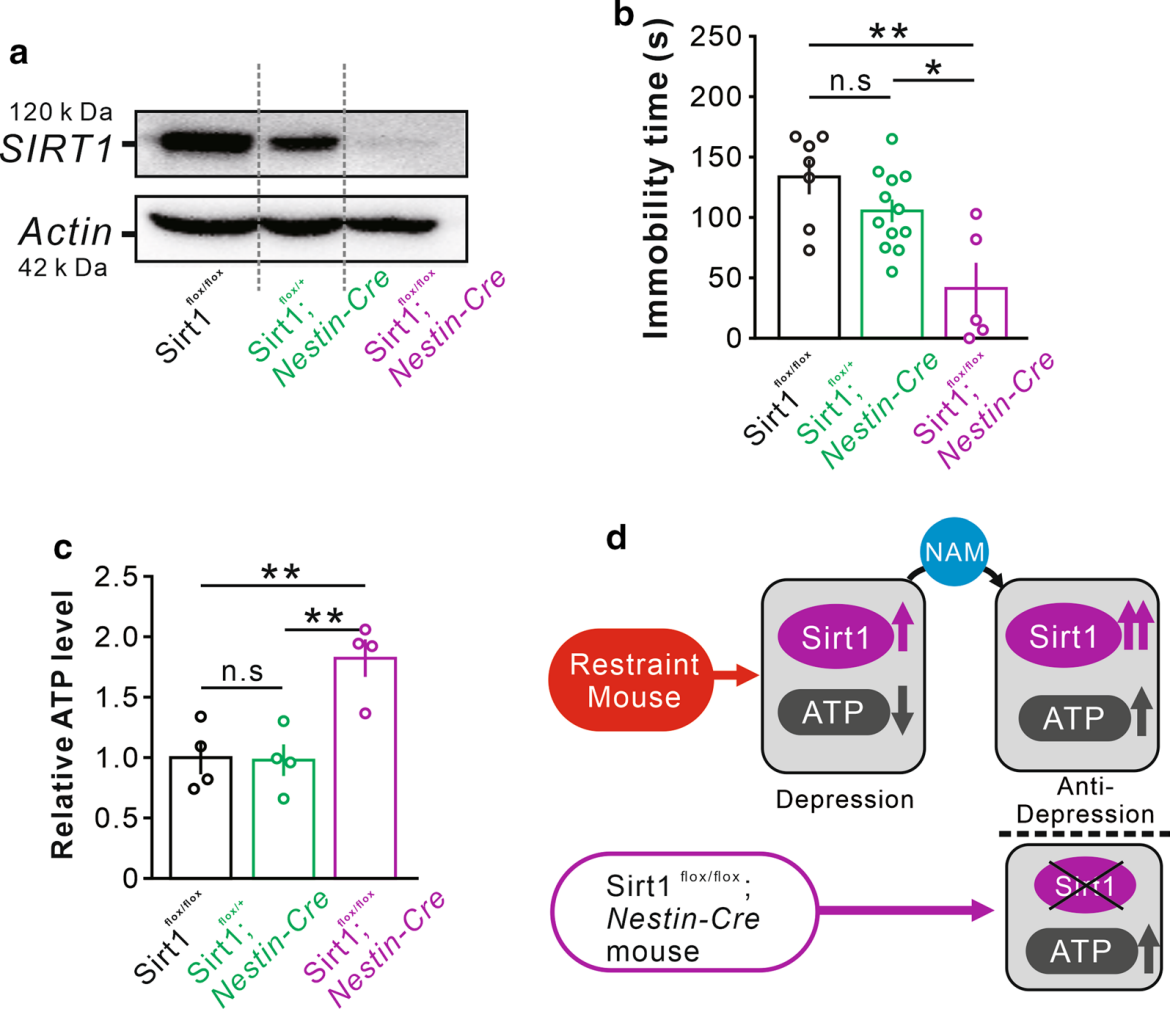
*Sirt1*^*flox/flox*^*; Nestin-Cre* mice exhibited antidepressant behaviors with increased ATP in the hippocampus. **a** Western blot was used to detect SIRT1 expression. **b** Immobility time in the forced swim test of different groups (n = 7/12/5 per group); *p < 0.05, **p < 0.01. **c** The relative of ATP level in the hippocampus (n = 4 per group); **p < 0.01. **d** Schematic representation of key molecules in the restraint model and *Sirt1*^*flox/flox*^*; Nestin-Cre* mice

Because the *Sirt1*^*flox/flox*^*; Nestin-Cre* exhibited antidepressant behaviors, we wondered if the phenotypes were related with ATP level when SIRT1 was absent. Western blot results showed efficient deletion of *Sirt1* in mice (Fig. 3a). The *Sirt1*^*flox/flox*^*; Nestin-Cre* mice exhibited reduced immobility times in the forced swimming test compared with *Sirt1*^*flox/flox*^ and *Sirt1*^*flox/*+^*; Nestin-Cre* mice (Fig. 3b). Accordingly, the ATP level in the hippocampus of *Sirt1*^*flox/flox*^*; Nestin-Cre* mice was significantly higher than that in *Sirt1*^*flox/flox*^ and *Sirt1*^*flox/*+^*; Nestin-Cre* mice (Fig. 3c).

As a summary (Fig. 3d), restraint depressive mice shown the higher SIRT1 activity and lower ATP level in the hippocampus, NAM increased the ATP level and SIRT1 activity, attenuated depressive-like behaviors. Besides, the level of ATP was increased in *Sirt1*^*flox/flox*^*; Nestin-Cre* mice with antidepressant behaviors. The findings indicated that the ATP played a vital role in the regulation of depression independent of SIRT1.

## Discussion

Clinical studies have demonstrated that NAM can stably improve the incidence of depression in patients, but the mechanism remains uncertain. NAM was previously thought to regulate a variety of physiological functions with the change of SIRT1. For example, Mitchell, S. J et al. found that chronic NAM supplementation could improve health span measures in mice without extending lifespans, and that enhanced acetylation of some SIRT1 targets in a diet and in NAM act in a dose-dependent manner [20]. However, Hwang et al. doubted the interpretation of results in studies that have used NAM as a SIRT1 inhibitor. They thought that NAM was an inhibitor of SIRT1 in vitro, while it could be a stimulator in cells [21]. Because SIRT1 activity was enhanced in the restraint depressive mice, we initially hypothesized that NAM could mediate depression by reducing SIRT1 activity. However, the application of NAM significantly reversed the depressive behaviors but increased SIRT1 activity further. These results showed that the change of SIRT1 activity was not consistent with the depressive phenotypes in mice. We also found that the *Sirt1*^*flox/flox*^*; Nestin-Cre* mice exhibited antidepressant behaviors, while *Sirt1* was deleted in brain. Combination with the discrepant changes of SIRT1 in depression, we speculated that SIRT1 could not play a direct role in the pathogenesis of depression.

Decreased ATP metabolism has been reported in patients with MDD and in animal models of depression [19, 22, 23]. Importantly, we found the level of ATP was reduced in the restraint model for depression, consistent with previous study [9], and recovered by the administration of NAM. As NAM could increase NAD^+^ levels that modulate the mitochondrial production of ATP through oxidative phosphorylation [12]. We concluded that NAM reduced depression-like behavior by increasing the ATP level in our mouse model. Besides, the further increased SIRT1 activity may also due to the raised NAD^+^ levels after NAM administration [21, 24, 25]. We also found that *Sirt1*^*flox/flox*^*; Nestin-Cre* mice were anti-depression with higher ATP level in the hippocampus of brain. However, the level of ATP in *Sirt1*^*flox/flox*^*; Nestin-Cre* mice resilient to 24-h-restraint stress need to be detected in the future. According to these results, we speculated that the level of ATP could regulate depressive-like behaviors, whether the SIRT1 activity was increased or deleted.

These results remind us that the role of the SIRT1-mediated pathogenesis of depression in the model of environmental stress must be reconsidered. This role may resolve some of the controversies surrounding the change of SIRT1 in depression. Our study also provides new insights into the use of NAM in treating depression.

## Data Availability

All data generated or analysed during this study are included in this published article.

## Abbreviations

NAM: Nicotinamide
SIRT1: Sirtuin 1
ATP: Adenosine triphosphate
MDD: Major depression disorder
NAD^+^: Nicotinamide adenine dinucleotide
CAC: Citric acid cycle
FST: Force swim test
SPT: Sucrose preference test
DG: Dentate gyrus
NAc: Nucleus accumbens
BSA: Bovine serum albumin
PVDF: Polyvinylidene fluoride
RT-PCR: Real-time polymerase chain reaction
SEM: Standard error of the mean
ANOVA: Analysis of variance
